# Supernatural Belief Is Not Modulated by Intuitive Thinking Style or Cognitive Inhibition

**DOI:** 10.1038/s41598-017-14090-9

**Published:** 2017-11-08

**Authors:** Miguel Farias, Valerie van Mulukom, Guy Kahane, Ute Kreplin, Anna Joyce, Pedro Soares, Lluis Oviedo, Mathilde Hernu, Karolina Rokita, Julian Savulescu, Riikka Möttönen

**Affiliations:** 1 Brain, Belief, & Behaviour Lab, Centre for Advances in Behavioural Science, Coventry University, Coventry, UK; 2 Philosophy Faculty, University of Oxford, Oxford, UK; 3 Department of Psychology, Massey University, Palmerston, New Zealand; 4 Faculty of Social and Human Sciences, Universidade Nova de Lisboa, Lisbon, Portugal; 5 Pontificia Universita Antonianum, Rome, Italy; 6 Institute of Cognitive and Culture, Queen’s University, Belfast, Ireland; 7 National University of Ireland, Galway, Ireland; 8 Department of Experimental Psychology, University of Oxford, Oxford, UK; 9 School of Psychology, The University of Nottingham, Nottingham, UK

## Abstract

According to the Intuitive Belief Hypothesis, supernatural belief relies heavily on intuitive thinking—and decreases when analytic thinking is engaged. After pointing out various limitations in prior attempts to support this Intuitive Belief Hypothesis, we test it across three new studies using a variety of paradigms, ranging from a pilgrimage field study to a neurostimulation experiment. In all three studies, we found no relationship between intuitive or analytical thinking and supernatural belief. We conclude that it is premature to explain belief in gods as ‘intuitive’, and that other factors, such as socio-cultural upbringing, are likely to play a greater role in the emergence and maintenance of supernatural belief than cognitive style.

## Introduction

What drives our belief in gods — intuition or reason; heart or head? There is a long debate in philosophy about the supposed irrationality or rationality of supernatural beliefs^1,2^ and, for over a century, sociologists have predicted the triumph of rationality and the disappearance of religion^3^. It has taken longer for psychologists to add to this discussion, but the last 20 years have seen some advances. The cognitive psychological literature advocates a direct link between intuitive thinking and belief in gods, by explaining that such beliefs are innate, pre-conscious, an experiential form of information processing, and the outcome of a hyperactive device of agency seeking^4,5^.

Intuitive thinking (associated with ‘System 1’ processing) involves rapid processing of information with little explicit deliberation. It is contrasted with analytical thinking (associated with ‘System 2’ processing) which is slower, deliberate, and effortful^6,7^. While System 1 is the default system of processing information, it can be overridden by System 2. Within this two-system framework, believing in the supernatural is considered to be an intuitive, non-rational form of information processing. This hypothesis suggests that supernatural beliefs are the result of an overgeneralization of a default system whereby the cause of events is attributed to supernatural agents, without looking for further explanations in an analytical way^4,8–10^. Thus, the suggestion is that while we have all cognitively evolved to be ‘born believers’^11^, the strength of supernatural belief may be predicted by how much individuals rely on intuitive versus analytic thinking^12^. We will refer to the proposal that supernatural belief is rooted in an intuitive form of thinking as the *Intuitive Belief Hypothesis*.

Experimental attempts to validate this hypothesis have focused less on intuition per se than on analytical thinking. For example, it has been shown that highly analytical thinkers have lower rates of supernatural beliefs than ‘intuitive’ individuals^13^. These and other studies, where experimentally increasing analytical thinking was associated with lower supernatural belief^14^, have been taken to support the view that to believe in gods and spirits is the natural outcome of the working of our intuitive system, and that this disposition can be overridden or corrected through analytical thinking.

There are, nonetheless, limitations to this approach, ranging from a lack of studies with ecological validity, the use of a culturally limited sample (mostly North American university students), to the overreliance on enhancing analytical rather than intuitive thinking, as it is generally assumed that an increase in analytical thinking means a concurrent decrease in intuitive thinking. It is not clear, however, whether the intuitive-analytical systems work together in a hydraulic-like way, so that an increase in one necessarily leads to a decrease in the other. On the contrary, it is possible that, despite some interactions, they work as ‘two minds in one brain’^15^. This might explain the evidence suggesting that supernatural beliefs might coexist with logical, scientific knowledge^16^, or why studies of tribal societies have depicted the existence of rational, instrumental thinking alongside supernatural ideas and rituals^17^. Likewise, cognitive studies have showed that believers in the supernatural and skeptics do not differ in their scientific knowledge about energy, though believers are able to hold both factual and supernatural information about energy as a ‘living or vital force’^18^. Other studies, using brain-imaging techniques, suggest that anatomically different areas of the brain are involved when one generates belief-based reasoning (the ventral medial prefrontal cortex) or logical reasoning (right prefrontal cortex)^19^.

In our first two studies, we investigated whether there was a relationship between supernatural beliefs and intuitive thinking in a way that adds to past approaches by broadening the population and methods used. To this end, we started by using an ecological prime for supernatural belief with a varied population, and tested its effects on intuitive thinking. Previous studies have relied primarily on undergraduate populations and supernatural primes that are far removed from everyday spiritual experiences. To overcome these limitations, we conducted a field study on the pilgrimage route to Santiago of Compostela that crosses most of northern Spain, a walk that takes around 30 days starting at the French Pyrenees. We chose this pilgrimage because it allowed for a measure of interaction with supernatural beliefs (length of time on pilgrimage), as well as having a particularly varied pool of participants, concerning religious affiliation and nationality. We used a probability bead game which allows for both analytical and intuitive responses. Participants were asked to draw a color bead from two containers, which had different quantities of colored and transparent beads. After the task, participants rated supernatural identification of religiosity and spirituality (‘How religious/spiritual do you consider yourself to be?’). Moreover, they reported the number of days they had been on pilgrimage. If the Intuitive Belief hypothesis is true, we expected that the length of time spent on pilgrimage would make participants more engaged with supernatural ideas, and thus increase intuitive thinking. Secondly, we expected pilgrims who identified more strongly as religious or spiritual to have higher intuitive thinking scores in the probability task.

In the second study we used a different paradigm from the dual-processing literature to examine the role of intuitive thinking in the endorsement of supernatural beliefs. We followed a reverse logic to that of the main Intuitive Belief Hypothesis literature, in that instead of enhancing analytical processing we aimed to deplete it by manipulating working memory load. We expected, if the Intuitive Belief Hypothesis were true, that by depleting analytical thinking we would enhance reliance on intuitive processing and, subsequently, strengthen supernatural beliefs.

If Systems 1 and 2 function with some degree of autonomy^15^ and believers are able to hold both naturalistic and supernatural explanations, we would expect these two studies to show no relationship between intuition and belief. If on the other hand, the Intuitive Belief Hypothesis is true, we would expect to find an association between intuition and belief.

We then turned our attention to analytical thinking, since the cognitive literature on belief attempts to enhance the functioning of this system. Previous authors have suggested that analytic processing may *inhibit* supernatural beliefs^14^. There is a large literature on cognitive inhibition, which has been defined as ‘the stopping or overriding of a mental process, in whole or in part, with or without intention’^20^. Much of the neuroimaging literature points to the right Inferior Frontal Gyrus (rIFG) as the key locus of inhibitory control^21^, and a recent study has showed a stronger activation of this brain area coupled with a decrease in supernatural attributions^22^. Since this association remains to be causally tested, in a third study we used brain stimulation to facilitate the rIFG in order to increase cognitive inhibition and measured whether this decreases supernatural beliefs.

## Study 1: The Way to Santiago

### Participants

Eighty-nine participants between the ages of 16 and 67 (*M* = 37 years, *SD* = 14 years), 55 female (61.8%) were recruited at pilgrim hostels where they stopped for rest and refreshment. Participants were spending on average 32 days on their pilgrimage (SD = 37 days, range = 5 to 354 days), and at the moment they took part in the study, they had been walking on average for 12 days (*SD* = 10 days, range = 1 to 49 days). Questionnaires and oral instructions were given in English, Spanish, or Portuguese. The pool of participants was, as expected, quite diverse regarding nationality: 34% were Spanish, 13% German, 12% American, 8% Brazilian, 4% South Korean, 3% Polish, and all other nationalities constituted less than 3% of the total each, including Italian, Irish, and French. Most participants (71%) were Christian, 20% were spiritual but not religious, 8% declared to be atheist, and 1% Buddhist.

## Results

An examination of responses across the four trials revealed that the analytical choice was made across all trials by 27% of participants, across 3 trials for 17%, across two trials for 28%, and just once for 12% of participants; 73% of participants made the intuitive choice at least once. These results were similar to those of the original study, where 82% of participants made the intuitive choice at least once^23^. The most intuitive choice was made once by 34% of the participants (choosing a 6% probability versus a 10% probability of drawing the colour bead). There were no significant correlations between the intuitive score on the game and the number of days participants had been on pilgrimage (*r* = −0.02, *p* = 0.86); controlling for religious/spiritual scores did not affect the correlation (*r* = −0.01, *p* = 0.91). Neither did we find a significant correlation between an intuitive score and supernatural identifications [religiosity (*r* = −0.13, *p* = 0.24); spirituality (*r* = −0.16, *p* = 0.15)].

## Study 2: Working Memory Load and Supernatural Attributions

### Participants

Thirty-seven participants (51% female) between the ages of 18 and 40 (*M* = 25 years, *SD* = 6 years) were recruited from the general public and the university. Participants provided informed written consent and were reimbursed £10 for their time.

#### Results

The experimental group (*M* = 1.06, *SD* = 1.00) scored significantly lower on the CRT than the control group (*M* = 1.95, *SD* = 1.17), *F*(1,35) = 6.14, *p* = 0.02, indicating that the experimental manipulation worked as expected, i.e., those in the working memory load condition gave more intuitive responses than those in control condition. However, we found no difference in strength of supernatural attributions between the working memory load (*M* = 1.75, *SD* = 0.63) and the control (*M* = 1.82, *SD* = 0.68) condition, *F*(1,35) = 0.12, *p* = 0.73. We also found no difference in the speed of making an attribution between the working memory load (*M* = 1118.12 ms, *SD* = 354.46 ms) and the control (*M* = 1313.21 ms, *SD* = 565.94 ms) condition, *F*(1,35) = 1.56, *p* = 0.22. We found no association between an intuitive thinking style and self-reported religiosity (*r* = −0.07, *p* = 0.67) or supernatural practices (*r* = −0.06, *p* = 0.72).

## Study 3: The Neural Enhancement of Cognitive Inhibition

### Participants

Ninety individuals (58.9% female), between the ages of 18 and 64 (*M* = 28 years, *SD* = 11 years) were recruited from the general public. We used the stop signal task (STT) as a standard measure of cognitive inhibition^24,25^ to verify an enhancement of this function during anodal stimulation. Previous studies have showed that anodal tDCS over the rIFG improves performance on this task^26–28^. Given individual variability in responsiveness to tDCS^29,30^, we selected tDCS sensitive individuals only. First, we computed the difference in reaction times between before and during anodal stimulation for every individual. Then, we created a group of individuals who showed a decrease in reaction time (reflecting improvement on the stop signal task) by selecting all individuals who had a difference score of <0 msec, indicating they became faster during anodal stimulation relative to pre-stimulation baseline. This group had forty-four participants (27 female), between the ages of 18 and 60 (age *M* = 30.98, *SD* = 12.52 years), including Christians (36.4%), Atheists (22.7%), Spiritual but Not Religious individuals (18.2%), Agnostics (18.2%), and Spiritualists (4.5%). These participants were significantly faster during stimulation in the anodal (*M* = 208.18 ms, *SD* = 45.13 ms) than in the sham condition (*M* = 233.56 ms, *SD* = 62.73 ms; *p* = 0.01). They also improved their performance relative to pre-stimulation baseline more in the anodal condition than in the sham condition, *t*(43) = −3.77, *p* < 0.001.

## Results

In the supernatural attributions task there was no change in attributions during anodal stimulation (*M* = 3.30, *SD* = 1.65) relative to sham stimulation (*M* = 3.44, *SD* = 1.60), *t*(43) = −1.20, *p* = 0.24. Similarly, in the implicit association task there was no change during anodal stimulation (*M* = 0.34, *SD* = 0.39) relative to sham stimulation (*M* = 0.32, *SD* = 0.42), *t*(43) = −0.26, *p* = 0.79. That is, although anodal stimulation facilitated cognitive inhibition in this selected group, it had no effect on supernatural beliefs or attributions.

Results on the CRT did not correlate with supernatural attributions under anodal or sham stimulation (*r*’s < −0.03, *p*’s > 0.57), nor with the implicit association task scores (*r*’s < 0.02, *p*’s > 0.71), or with religiosity (*r* = 0.11, *p* = 0.47), or spiritual practices (*r* = 0.14, *p* = 0.38).

## General Discussion

We set out to carry three new studies to test the Intuitive Belief Hypothesis. Using a pilgrimage setting as an ecological prime for the supernatural, we found no evidence that the number of days spent on pilgrimage was associated with an intuitive thinking style. More importantly, we also found no indication that an intuitive response was positively associated with supernatural identifications. We used a much more diverse sample than previous studies with respect to nationality, which may explain the different results from previous studies. However, this field study had the limitation of not having a control group.

The next study used a standard randomized procedure of participants into experimental and control group, and instead of manipulating belief we attempted to increase intuitive thinking and included supernatural belief as the outcome variable. Although the working memory load manipulation led to more intuitive responses, when compared to a control condition, it still had no effect on how much, or how fast, one endorsed supernatural attributions.

In the third study, we applied brain stimulation to enhance cognitive inhibition, a process previously suggested to down-regulate supernatural belief^22^, and we again observed no changes in two belief measures. Moreover, we found no association between a standard test of dual processing thinking and any of the belief measures, which reinforces the results of our first two studies casting doubt on the Intuitive Belief Hypothesis.

There may be various explanations for our finding that supernatural belief is not associated with intuitive thinking nor influenced by manipulations that are expected to influence such System 1 processing. One possibility is our use of a varied general population and a different cultural context. While the majority of previous studies used US participants, and predominantly students, ours were conducted with British and other European participants. Sociological surveys have shown that Europe, including Britain, is less religious than the US^31^. It is likely that being religious in a secular culture is more cognitively demanding, as you are challenged to articulate the arguments for holding supernatural beliefs in a way that you aren’t in a society where the default is to be religious. This possibility is indirectly corroborated by surveys showing that in the UK religious people under 35 are better educated than the non-religious^32^, unlike in the US^31^. It is, thus, possible that supernatural belief might be associated, to a modest degree, with intuitive thinking in some cultural contexts (e.g., Christianity in the US) but not in others. Overall, the socio-educational upbringing is likely to play a much larger role in the strength of supernatural belief than cognitive styles and biases^33^.

Another possibility is that the Intuitive Belief Hypothesis is fundamentally wrong. Its key assumption that belief in the supernatural is the natural by-product of ordinary cognition does not easily explain the hundreds of millions of people who reject such beliefs. The related hypothesis that non-believers are able to cognitively inhibit the default tendency to believe is also unlikely from an evolutionary perspective, as it would require too much cognitive effort to continuously suppress supernatural ideas and attributions. In this respect, we should note that there are other recent failed experiments of the larger ‘naturalness of religion’ theory. For example, the idea that we are compelled to see supernatural agents because we possess an innate perceptual ‘hyperactive agency detection device’^1,5^ has been tested and disconfirmed across various experiments^34^.

How do supernatural beliefs arise? These are possibly the earliest kind of structured human beliefs as evidenced by archaeological findings of how dead bodies were carefully disposed of as early as the Neanderthals^35^. There is ample evidence that supernatural beliefs play an important function in meaning-making, emotional compensation, and potentially in the modulation of physiological responses^36–38^. They fulfill a need to predict and perceive the world, and to reduce uncertainty in the environment by generating ideas about the ultimate structure of the world and how to act in it^39^. But this doesn’t necessarily imply that we are ‘born believers’ in the way we inevitably learn a language at an early age. What is actually suggested by the wealth of sociological, historical, and other data is that whether one has strong supernatural beliefs or none at all is primarily based on social and educational factors^31,32,40^ and not on core cognitive dispositions.

What drives our belief in the supernatural, heart or head? There is a long historical debate on this matter. William James famously framed religion as an experiential, emotive process^41^. He did so, in part, because he was trying to establish the study of religion as a psychological endeavour, instead of a philosophical or theological one — he called theology “the metaphysical monster which… is an absolutely worthless invention of the scholarly mind”. But his work also considers the intellectual ideas associated with religion, and trying to nail supernatural belief as either intuitive or analytical, a matter of the heart or head, would for James be a simplistic solution.

The very idea that belief is natural is historically rooted in an attempt by early modern scientists to find God in nature^42^. Although the scientific methods we used have changed, some of the ideas keep coming back in different guises. Our studies here suggest that it is probably about time psychologists reconsider their understanding of belief as ‘natural’ or ‘intuitive’, and instead focus on cultural and social learning factors that give rise to supernatural ideas. Religious belief may be rooted in our society and culture (a sociocultural ‘meme’), rather than in some primitive gut intuition.

## Methods

All of the studies received ethical approval by ethical committees from Oxford University and Coventry University and they were conducted in accordance with relevant guidelines and regulations. All participants confirmed their informed consent to take part in the studies. The datasets generated and analyzed during the current studies are available from the corresponding author on request.

### Study 1

**Figure 1:**
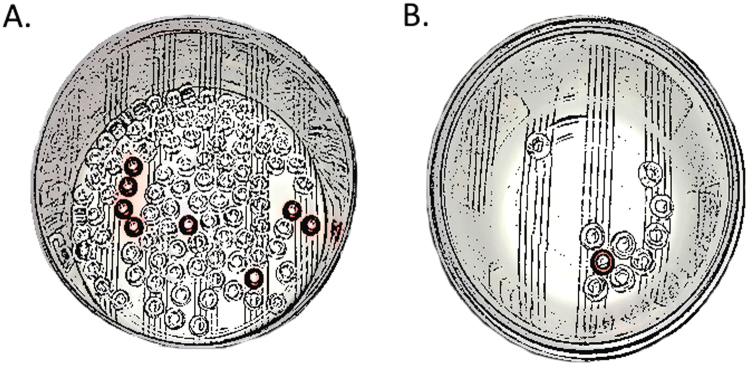
Illustration of the two bowls participants had to draw from while blindfolded. The task was to decide, before being blindfolded, where to attempt drawing a color bead from. The smaller bowl (**B**) always presented a 10% probability of getting the color bead, while the larger bowl (**A**) varied between a 6–9% probability.

Participants were presented with two different bowls containing a number of transparent and color beads^23^. The smaller bowl always presented a 10% probability of getting the color bead (one out of ten beads), while the larger bowl varied between a 6-9% probability (six to nine out of 100 beads; see Fig. 1). The probabilities in the large bowl were varied according to a Latin Square design (examples of two sequences of presentations of color beads were: 6-9-8-7 and 7-6-8-9). Participants were explicitly told the odds of the color to the transparent beads in each bowl, and were then asked to choose one to try drawing the color bead from. After they made their choices, we used a dark cloth to hide the contents of the bowl from the individual and shook the bowl to move the beads. There were 4 trials for each participant. Each participant was offered a chocolate bar for taking part in the study.

To score the trials, we assigned a score of 0 to the analytical choice, a score of 1 to a 9% probability of drawing the color bead from the large bowl, 2 to an 8% probability, 3 to a 7% probability, and 4 to a 6% probability. The smaller bowl was always the analytical choice, but intuitively one may have felt more inclined to choose the larger bowl, as it presented a greater number of color beads (a number of participants reported this in the original study and this was replicated in our study). Thus, a higher score indicated a more intuitive response.

### Study 2

Working memory can be defined as the virtual workspace where several mental items are juggled simultaneously as various operations are performed, and it can attend to a limited number of items (seven, minus or plus two^43^). ‘Loading’ working memory, by keeping more information in mind than one’s capacity can hold, has been shown to interfere with analytical processing, leading individuals to rely more on holistic, associative judgments, associated with actions that would ‘tempt fate’^40^. However, the actions in this study were not specifically related to supernatural beliefs.

In Study 2, we tested the Intuitive Belief Hypothesis by manipulating intuition via working memory load and measuring its impact on level of supernatural attributions. Participants were randomly assigned to the experimental working memory load or control tasks. We used two outcome measures: the Cognitive Reflection Test (CRT)^44^, which involves three mathematical puzzles designed to measure the overriding of an intuitive (incorrect) response; and the Supernatural Attribution task^22^, which was used to test the strength and speed of supernatural attributions. After completing the Supernatural Attribution Task, participants completed the CRT and supernatural belief scale measures. In line with the Intuitive Belief Hypothesis, we expected the working memory load condition to lead to stronger and faster evaluations of supernatural attributions, and a general intuitive thinking style to positively correlate with supernatural belief.

Participants either completed an experimental task or control task. In the experimental condition, working memory load was stimulated with a mental subtraction task. Participants were given a three-digit starting number at the beginning of the task and were asked to subtract three for each subsequent trial, and enter the updated number in a random 33% of trials. This percentage was chosen to make the mental subtraction task more difficult, as more frequent opportunities to provide the updated number would be less taxing to working memory capacity. The control version of the trial required participants to re-enter a three-digit number seen on the screen when prompted in a random 33% of trials. The control task sought to emulate the numeric and motor responses given by participants in the experimental condition, but without the working memory load. Measurement of the working memory load manipulation was reflected in number of correct responses in the two groups during the 33% random trials.

The Cognitive Reflection Test^44^ was used as a measure of intuitive-analytical thinking and allowed for a manipulation check, to confirm that the working memory load manipulation would indeed change levels of intuitive thinking. An example of a question on the CRT is: “A bat and a ball cost $1.10 in total. The bat costs $1.00 more than the ball. How much does the ball cost?”. The Supernatural Signs Task consisted of 30 story–picture pairs based on life changing events^22^. Participants were asked to evaluate each story-picture pair using a four-point scale (1—strongly disagree, 2—disagree, 3—agree, 4—strongly agree): ‘If I was in the situation described and saw [that poster/picture], I would think the picture contained a sign or a message about how [the situation] will turn out.’ Higher evaluations indicated the participant agreed to the existence of a supernatural attribution (i.e. seeing a sign or a message in the image). Reaction times were recorded for all evaluations. An example of a story reads: ‘You have lost your home in a fire and are afraid that the refund from your insurance company will be inadequate’ followed by a picture of a park bench. Participants were given the following instructions: ‘You will be shown stories and a picture after each story. Imagine you are walking down the street and are deep in thought, thinking about the situation described in the story. Suddenly you see the picture on a large poster right in front of you. Try to think about what thoughts the picture might raise in you in that situation.’ Finally, to assess endorsement of the supernatural, a one-item measure of religiosity (‘How religious do you consider yourself to be?’) and a three-item scale of frequency of supernatural practices (prayer, attending religious services and reading religious books) were used.

### Study 3

Neurocognitive research on cognitive inhibition, including imaging and brain lesion studies, has shown that rIFG impairments compromise performance on Go/No Go tasks and on syllogistic reasoning tasks^21,45^. If cognitive inhibition is the key process responsible for modulating belief, we can reconsider the usefulness of tying it to a general dual processing system, particularly given the failure in finding an association between intuition and belief in our first two studies.

In this third study, we aimed to manipulate cognitive inhibition by using anodal transcranial direct current stimulation (tDCS) to enhance activity in the rIFG^26–28^. Participants had the tDCS electrodes placed and then completed a Stop Signal Task before and during anodal and sham stimulation, to measure levels of cognitive inhibition. This task was followed by a Supernatural Attribution task^22^ and an Implicit Association Belief task (IAT;^46^) to measure levels of supernatural belief during anodal and sham tDCS. Before coming into the stimulation sessions, participants completed the CSR and short measures of supernatural belief. Our general hypothesis was that by enhancing cognitive inhibition we would down-regulate supernatural beliefs.

We carried out a double-blind experiment using anodal and sham (placebo) tDCS (NeuroConn, Germany), applied over the rIFG during the experimental/behavioral tasks. Each participant attended both anodal and sham sessions, which were separated by one week. Both experimenters and participants were blind to the type of stimulation being used in each session (anodal vs. sham). The order of the sessions was counterbalanced across participants. A direct current of 1 mA was applied using two saline-soaked surface sponge electrodes (7 × 5 cm). In the anodal session, stimulation was ramped up in the initial 15 seconds and held constant for 20 min. In the sham session, stimulation was ramped up in the initial 15 seconds, held constant for 15 seconds, and ramped down for 15 seconds. The anode electrode was placed over the rIFG and the cathode electrode over the left supraorbital ridge. Localisation was established using the 10–20 EEG system with the rIFG as the cross-point between T4-Fz and F8-Cz^47^. All tasks were presented on a 23-inch computer screen with a 1920 × 1080 resolution 90 cm in front of the participant. C++ was used to present the stop signal task. All other tasks were presented in E-Prime professional (version 2.0).

We used the stop signal task (STT) as a standard measure of cognitive inhibition^24,25^ to verify an enhancement of this function during anodal stimulation. Previous studies have showed that anodal tDCS over the rIFG improves performance on this task^26,27^. The STT requires participants to classify a stimulus by pressing a computer key and to withhold their response on a random selection of 25% of trials following an auditory ‘beep’ (759 Hz) lasting for 75 ms. The program we used^25^ adjusted the stop-signal delay (SSD) after every stop-signal trial and started with a delay of 250 ms. Following successful stopping, it increased in 50 ms increments, and following unsuccessful stopping the delay was decreased by 50 ms increments. The task consisted of two blocks of 128 trials with a 10 second break between trials. Visual stimuli were 1.5 cm in diameter and were presented in white in the centre of a black background for 1250 ms with an inter-stimulus interval of 2000 ms.

**Figure 2:**
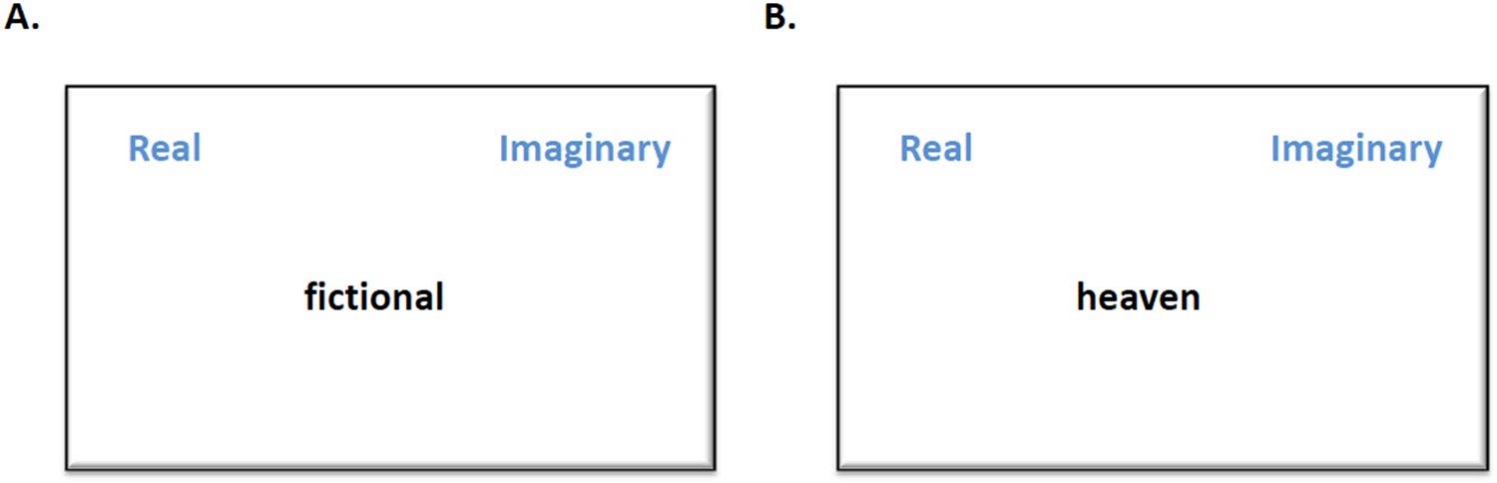
Examples of stimuli from the IAT. The ‘real’ category was always presented on the left, and associated with the ‘e’ key, while the ‘imaginary’ category was on the right, associated with the ‘i’ key. In all blocks, the presented ‘real’ and ‘imaginary’ words thus had to be associated with their category on the left and right respectively (**A**). As for the presented religious/spiritual words, participants had to associate religious/spiritual stimuli with the left, ‘real’ category in the first test block, and with the right, ‘imaginary’, category in the second test block (**B**).

The Supernatural Attribution Task used in this study is the same task as described in Study 2, where participants read a story followed by a picture and were asked to attribute whether the picture contained a ‘sign’ as to how the story would unfold, except that we adapted the rating scale to 8 points (1 = strongly disagree to 8 = strongly agree). The stimuli were divided into two sets so that participants were presented with 19 picture-story pairs in each session. Participants completed a single-target Implicit Association belief test (adapted from^46^), which measured the speed and accuracy of associating religious and spiritual words with the categories ‘Real’ and ‘Imaginary’ (see Table 1). This consisted of one training block and two test blocks. In the first test block, ‘real’ and ‘religious/spiritual’ stimuli were associated and categorized to the left using the ‘e’ key, whilst ‘imaginary’ words were categorized to the right using the ‘i’ key (see Fig. 2). The second test block was reversed so that ‘religious/spiritual’ stimuli were categorized to the right, using the ‘i’ key, which means they were paired with the ‘imaginary’ category. Stimuli appeared in a random order with an inter-stimulus interval of 500 ms. All ‘imaginary’ and ‘real’ stimuli were presented three times, while religious/spiritual stimuli were presented twice in each block to ensure an equal number of correct keystrokes to the left and right. We used Greenwald’s IAT scoring algorithm^48^ to process the results of the implicit association belief task (see below). The order of presentation of the computer tasks (Supernatural Attribution and Implicit Association Belief tests) was randomized between participants but, in order to control for the effects of fatigue, each participant completed the tasks in the same order across both stimulation sessions.

**Table 1:** Stimuli presented in the Implicit Association Belief test.

Real	Imaginary	Religious	Spiritual
fact	fake	hell	aura
true	myth	soul	ghost
valid	hoax	devil	karma
actual	bogus	angel	chakra
proven	untrue	demon	6th sense
genuine	unreal	heaven	telepathy
physical	illusion	holy spirit	poltergeist
authentic	fantasy	resurrection	reincarnation
legitimate	fictional	Adam and Eve	supernatural

For the supernatural attribution task, a paired samples t-test was carried out on the dependent variable *supernatural attributions* with stimulation condition as independent variable (anodal and sham). For the implicit association belief task, the D-scores were entered as a dependent variable into a paired samples t-test with stimulation condition (anodal and sham) as independent variable. The IAT task was analysed in accordance with Greenwald’s scoring algorithm^48^. The practice block was excluded from the analysis, leaving two test blocks. We monitored whether participants had responses faster than 300 ms in 10% of their trials, but did not need to exclude any participant. Responses slower than 10,000 ms from stimulus onset were excluded from the analysis (9 trials, 0.03%). Incorrect responses were given an error penalty; these responses were replaced by the mean of the block plus 600 ms. D-scores were determined by first calculating the standard deviation of the two test blocks together. Finally, the difference between test block 1 and 2 was computed by subtracting test block 2 from test block 1, such that a positive score indicated that religious/spiritual words were implicitly regarded as imaginary, and a negative score as real. Finally, this difference score was divided by the standard deviation.
